# The impact of heat inactivation on RT-qPCR detection of severe acute respiratory syndrome coronavirus 2 (SARS-CoV-2): an experience from the University Clinical Centre of Vojvodina, Serbia

**DOI:** 10.2478/aiht-2025-76-3973

**Published:** 2025-12-30

**Authors:** Jelena Stojčević Maletić, Iva Barjaktarović, Ljiljana Andrijević, Katarina Bačulov, Slobodanka Bogdanović Vasić, Diandra Pintać Šarac

**Affiliations:** University of Novi Sad Faculty of Medicine, Novi Sad, Serbia; University Clinical Centre of Vojvodina, Novi Sad, Serbia; Šabac Academy of Vocational Studies, Šabac, Serbia

**Keywords:** COVID-19, GeneFinder nucleic acid test, human *RNase* P gene, nasopharyngeal swabs, oropharyngeal swabs, quantification cycle, real-time quantitative polymerase chain reaction, RNA isolation, gen za humanu RNazu P, GeneFinder test nukleinskih kiselina, izolacija RNA, kvantifikacijski ciklus, kvantitativna lančana reakcija polimerazom u stvarnom vremenu, nazofaringealni brisovi, orofaringealni brisovi

## Abstract

Handling clinical samples from patients suspected of SARS-CoV-2 infection puts healthcare workers at risk of exposure to infectious particles. To reduce this risk, samples are often heat-inactivated before nucleic acid isolation, but this procedure may affect the analytical sensitivity of the test. The aim of this study was therefore to evaluate the effects of heat inactivation (56 °C for 30 min) on RT-qPCR results of samples taken from nasopharyngeal and oropharyngeal (NP/OP) swabs collected from 200 symptomatic patients. Each sample was split into two aliquots – one subjected to heat inactivation and the other stored at 4 °C – followed by nucleic acid isolation and RT-qPCR analysis using the GeneFinder COVID-19 nucleic acid test. Heat inactivation did not significantly affect the overall SARS-CoV-2 detection rate (55.5 % vs. 55.0 % in untreated and heat-treated groups; χ^2^=0.01; p=0.91). However, discrepancies occurred in 15.3 % of samples, all with quantification cycle (Cq) values >31, including target loss, gain, or complete signal disappearance after heat treatment. Heat inactivation also slightly decreased Cq values for the RNA-dependent RNA polymerase (*RdRp*) and envelope (*E*) genes and increased those for the nucleocapsid (*N*) gene, with significant changes in strongly positive samples (Cq≤33). In positive samples (Cq≤40), the human ribonuclease (*RNase*) P gene also exhibited significantly higher Cq values after heat treatment. In the strongly positive subgroup, correlation analysis showed moderate correlation for *RdRp* and very strong correlation for the *N* and *E* genes, and a weaker correlation for weakly positive samples. In conclusion, heat inactivation at 56 °C for 30 min does not significantly affect viral gene detection but may diminish it in samples with low viral load.

## Abstract (hr)

Rukovanje kliničkim uzorcima pacijenata sa sumnjom na infekciju virusom SARS-CoV-2 potencijalni je rizik od izloženosti infektivnim česticama među zdravstvenim djelatnicima. Toplinska inaktivacija uzoraka prije izolacije nukleinskih kiselina može poboljšati biosigurnost tijekom testiranja, ali i utjecati na analitičku osjetljivost testa. Ovim je istraživanjem procijenjen učinak toplinske inaktivacije (56 °C tijekom 30 minuta) na rezultate RT-qPCR analize brisova nazofarinksa i orofarinksa (NF/OF), prikupljenih od 200 simptomatskih pacijenata. Svaki je uzorak podijeljen u dva alikvota: jedan je podvrgnut toplinskoj inaktivaciji, a drugi pohranjen na 4 °C, nakon čega su provedene izolacija nukleinskih kiselina i RT-qPCR analiza korištenjem GeneFinder COVID-19 testa nukleinskih kiselina. Toplinska inaktivacija nije značajno utjecala na ukupnu stopu detekcije SARS-CoV-2 (55,5 % prema 55,0 % u netretiranim i toplinski tretiranim skupinama; χ^2^=0,01, p=0,91). Međutim, razlike su zabilježene u 15,3 % uzoraka, svi s vrijednostima kvantifikacijskog ciklusa (Cq)>31, uključujući gubitak ili pojavu cilja te potpuni nestanak signala nakon tretmana. Toplinska inaktivacija blago je smanjila vrijednosti Cq za *RdRp* i *E* gene, a povećala za *N* gen, uz značajne promjene u skupini visokopozitivnih uzoraka (Cq≤33). U skupini pozitivnih uzoraka (Cq≤40), gen za humanu Rnazu P također je pokazao značajno više vrijednosti Cq nakon toplinskog tretmana. U podskupini visokopozitivnih uzoraka, analiza korelacije pokazala je umjereno slaganje za *RdRp*, vrlo snažnu korelaciju za *N* i *E* te slabiju podudarnost u niskopozitivnim uzorcima. Zaključeno je da toplinska inaktivacija na 56 °C tijekom 30 minuta ne utječe značajno na detekciju virusnih gena, ali ju može smanjiti u uzorcima s niskim virusnim opterećenjem.

On 11 March 2020, the World Health Organization (WHO) declared the coronavirus disease 2019 (COVID-19) a global pandemic (1, 2). By June 2024, about 776 million cases of SARSCoV-2 infection and seven million deaths had been documented worldwide (3). Laboratory COVID-19 testing was crucial for establishing the incidence of infection as precisely as possible, but due to the limited time window, the WHO forewent serological assays or the nucleic acid amplification tests and instead recommended the use of amplification of viral ribonucleic acid (RNA) by reverse transcription-quantitative real-time polymerase chain reaction (RT-qPCR) as the gold standard for diagnosis (4,5,6,7,8,9). RT-qPCR can identify several genes encoding viral structural proteins, such as the spike (*S*), envelope (*E*), membrane (*M*), and nucleocapsid (*N*) (10), along with eight accessory proteins. It can also detect the open reading frame-1 (*ORF1ab*) gene, which encodes non-structural polyproteins which are cleaved to encode 16 non-structural proteins (NSP1–16) responsible for viral transcription and replication, including the RNA-dependent RNA polymerase (*RdRp*, NSP12) (11, 12).

Guidelines regarding which genes to target for SARS-CoV-2 detection vary worldwide. During the pandemic, the WHO recommended protocols targeting the *E* gene for initial screening and the *RdRp* gene for verification (6). The US Centers for Disease Control and Prevention (CDC) reported that the newly developed 2019-nCoV RT-qPCR test targeted multiple regions of the *N* gene (13). In the developed SARS-CoV-2 RT-qPCR assays, the *N* gene is the most commonly chosen target alongside *ORF1ab*, while the *S* gene is the least frequent target. Given the potential genetic variability of SARS-CoV-2, RT-qPCR assays targeting multiple genomic regions are recommended to improve diagnostic reliability and reduce the likelihood of false-negative results (14).

Considering the risks of infection, molecular testing of clinical specimens must be carried out in a laboratory that meets the minimum biosafety level 2 (BSL-2) requirements (15), and all specimens should undergo their first sample processing in a proper biosafety cabinet, but since such cabinets may not be available in specific situations, the alternative to go forward with RNA isolation is to inactivate the SARS-CoV-2 virus beforehand to prevent COVID-19 transmission among medical staff. There are several and physical (16, 17) and chemical (18, 19) ways to inactivate SARSCoV-2, and heat is the most common choice at varying temperatures and timings, such as 56 °C for 30 min, 60 °C for 60 min, 92 °C for 15 min, 80 °C for 5 min, or 100 °C for 1 min (20,21,22).

However, heat treatment may degrade viral RNA and result in false-negative findings (23). The aim of this study was to look further into this issue by assessing how exactly heat inactivation affects the detection of SARS-CoV-2 in the hope that our findings could inform future research and inactivation approaches prior to nucleic acid isolation for COVID-19 testing.

## MATERIALS AND METHODS

For this purpose we collected 200 clinical specimens from 200 randomly selected adult patients suspected of COVID-19 who were hospitalised at the University Clinical Centre of Vojvodina (UCCV) in Novi Sad, Serbia between 11 and 19 November 2023. The study was approved beforehand by the hospital’s Ethics Committee on 24 February 2023 (approval No. 00-51).

We took one nasopharyngeal (NP) and one oropharyngeal (OP) swab from each patient and placed them in the same tube with 3 mL of viral transport medium treated with antibiotics and antifungal agents (Cat. No. SL901B, Lot No. 200405, SANLI Medical Technology Development Co., Liuyang, Hunan, China). In other words, each patient gave one combined NP/OP sample. Samples were then transported to the UCCV Virology Laboratory, and the tertiary containers in which they were transported opened under the BSL-2 conditions, in accordance with the WHO standards (24).

A 1000 µL NP/OP sample was divided into two sterilised cryogenic tubes. One 500 µL aliquot was heat-treated at 56 °C for 30 min in a thermoblock (ThermoMixer^™^ F1.5, Eppendorf, Hamburg, Germany) and then stored at 4 °C until analysis, while the second, control aliquot was not heated but only stored at 4 °C. Every further step, including isolation and master mixing, was identical and followed the standard operating procedure to minimise variables that could affect the results. Within 12 h of collection, all 200 NP/OP samples (200 in the heat-treated batch and 200 in the matching control batch) were tested in duplicate (two technical replicates) as described below.

### RNA isolation and RT-qPCR analysis

Viral RNA was isolated using the GeneRotex 96 Nucleic Acid Extractor (Xi’an Tianlong Science and Technology Co., Ltd., Xi’an City, China) in combination with the Viral DNA and RNA Extraction Kit (Cat. No. T014H, Lot No. 23081710T014H, Xi’an Tianlong Science and Technology) following the manufacturer’s instructions (25). The isolation procedure is based on magnetic bead technology, which enables adsorption, transfer, and release of nucleic acids, combined with a proprietary buffer system for lysis, binding, washing, and elution. A 200-μL aliquot of viral transport medium was processed per sample, and nucleic acids were eluted in 80 μL of elution buffer. The isolated RNA showed sufficient purity and yield for downstream RT-qPCR analysis, as confirmed by successful amplification of the internal control (IC) gene. According to the manufacturer’s specifications, this extraction kit allows recovery of viral DNA from samples containing more than 10 IU/mL and viral RNA from samples containing more than 30 IU/mL (25).

Preparation of reaction mixtures, amplification, and detection were performed on a Gentier 96E RT-qPCR system (Xi’an Tianlong Science and Technology) using the GeneFinder^™^ COVID-19 Plus Real*Amp* kit (Cat. No. IFMR-45, Lot No. 2006-R, OSANG Healthcare Co., Ltd., Anyang, Korea) and following the manufacturer’s instructions (26). Each 20 µL reaction mixture contained 10 µL of COVID-19 Plus Reaction Mixture (including Taq DNA polymerase, reverse transcriptase, dNTPs, Tris-HCl, and MgCl_2_), 5 µL of COVID-19 Plus Probe Mixture (specific primer pairs and probes for amplification and detection of each target), and 5 µL of isolated RNA. Thermal cycling conditions included reverse transcription at 50 °C for 20 min, followed by pre-denaturation at 95 °C for 5 min, 45 cycles of denaturation at 95 °C for 15 s, and another 45 cycles of annealing/extension at 58 °C for 60 s.

Real-time detection targeted three SARS-CoV-2 genes, namely *RdRp*, *E*, and *N*, while human *RNase* P gene served as IC for sample adequacy and isolation efficiency. Carboxylfluorescein (FAM) signals were recorded for the *RdRp* gene, 4,5-dichloro-dimethoxyfluorescein/2′-chloro-7′-phenyl-1,4-dichloro-6-carboxyfluorescein (JOE/VIC) signals for the *N* gene, sulforhodamine 101 acid chloride (Texas Red) signals for the *E* gene, and cyanine 5 (Cy5) signals for the human *RNase* P gene, and their quantification cycle (Cq) values automatically recorded by the instrument’s software.

A sample was considered positive for SARS-CoV-2 if at least one of the viral target channels (FAM, JOE/VIC, or Texas Red) yielded a sigmoidal amplification curve with Cq≤40, regardless of whether IC amplification confirmed it (Cq≤40) or not (IC Cq>40). A sample was regarded negative if no amplification of the target gene was detected, even though IC was successfully amplified. The results were deemed invalid if neither the targets nor IC were amplified, indicating possible isolation failure, PCR inhibition, or poor sample quality. Such samples were re-isolated and retested.

The GeneFinder kit includes three controls – positive (PC), negative (NC), and IC – to verify the performance of reagents and assay procedures. Positive and negative controls are included in each RT-qPCR run to confirm amplification efficiency and to monitor potential contamination. The positive control contains four non-infectious DNA plasmids encoding the *RdRp*, *E*, *N*, and *RNase* P genes and must produce a sigmoidal amplification curve in the FAM, Texas Red, JOE/VIC, and Cy5 channels at Cq values ≤22 for the RdRp, E, and N genes, and Cq≤21 for *RNase* P (IC). The negative control, which is diethyl pyrocarbonate (DEPC)-treated water, should show no amplification curve in any of the channels (no Cq or Cq>40).

### Statistical analysis

All data were analysed using the IBM^®^ SPSS Statistics version 23.0 (IBM SPSS Inc., Armonk, NY, USA). Cq values were compared between the heat-treated and control batches of samples with the McNemar’s chi-squared test and Pearson correlation coefficient. Considering that the data were distributed normally, we used the paired *t*-test to determine mean differences in Cq values between the two batches. A p-value <0.05 was considered statistically significant.

## RESULTS

Overall, 111 (55.50 %) control and 110 (55.00 %) heat-treated samples tested positive. Table 1 shows the distribution of SARSCoV-2 gene positivity patterns. Positive tests to all three genes dominated in both control and the heated batches, but 17 (15.30 %) samples showed different test results for target genes, all with Cq values >31 (Table 2). These included seven samples (6.30 %) that lost one or more positive target genes after heat treatment, one sample (S 028; 0.90 %) that became completely negative after heat treatment, and three (2.70 %) samples that gained a positive target gene after heat treatment.

The McNemar chi-squared test showed no significant difference in the proportion of positive and negative results between the matching heated and control batches (χ^2^=0.01, p=0.91).

However, heat inactivation did affect Cq values of the target genes (Table 3). In the strongly positive subgroup (Cq≤33), heat treatment slightly lowered the mean Cq of the *RdRp* and *E* genes, while the mean Cq of the *N* gene was significantly higher than in the control samples.

In addition, heat treatment in positive samples (Cq≤40) affected the Cq values for the human *RNase* P gene, which were significantly higher than in the control batch.

**Figure 1:**
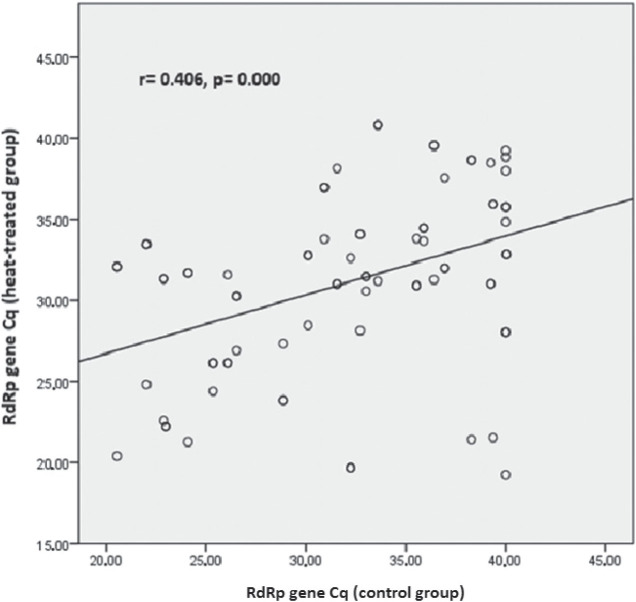
Correlation between the *RdRp* gene Cq values in the control and heat-treated strongly positive (Cq≤33) sample batch

**Figure 2:**
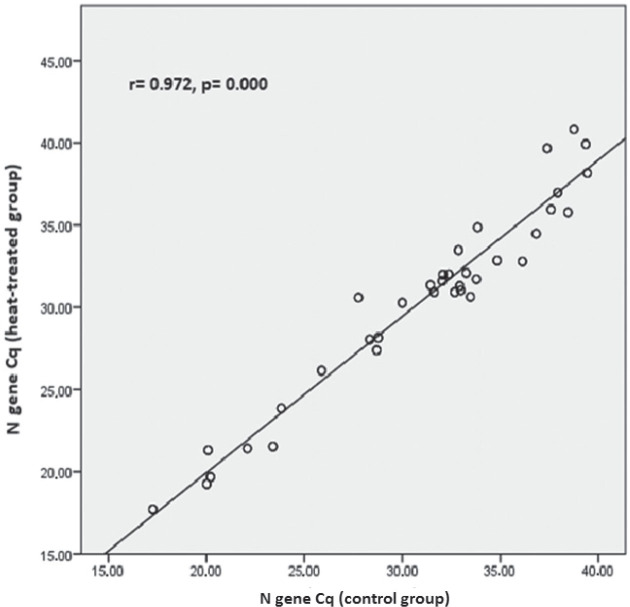
Correlation between the *N* gene Cq values in the control and heat-treated strongly positive (Cq≤33) sample batch

**Figure 3:**
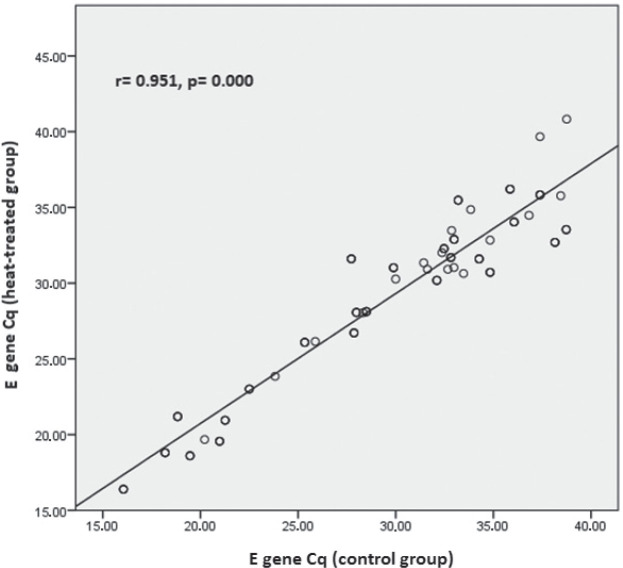
Correlation between the *E* gene Cq values in the control and heat-treated strongly positive (Cq≤33) sample batch

**Figure 4:**
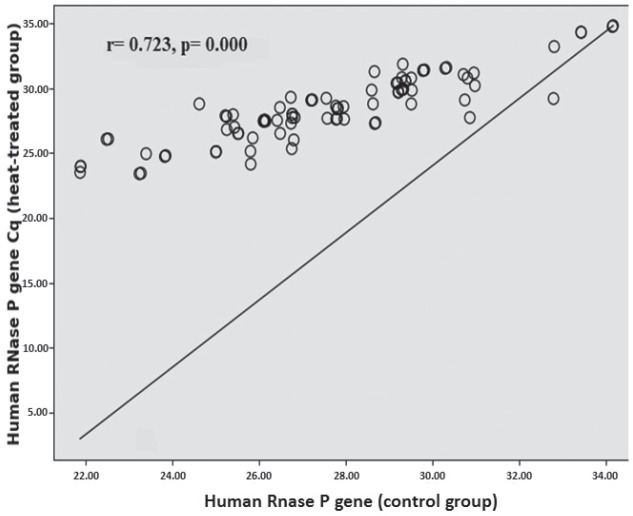
Correlation between the human *RNase* P gene Cq values in the control and heat-treated positive sample batch

In the weakly positive subgroup (Cq>33), only the *E* gene had significantly lower Cq values (p=0.020) than control. In addition, in the strongly positive subgroup correlation analysis revealed moderate correlation between heat-treated and control samples for *RdRp* (r=0.406; p=0.000) (Figure 1) and a very strong correlation for the *N* and *E* genes (r=0.972; p=0.000 and r=0.951; p=0.000, respectively) (Figures 2 and 3). In positive samples (Cq≤40), correlation analysis revealed a strong correlation between heat-treated and control batches for the human *RNase* P gene (r=0.723; p=0.000) (Figure 4). In the weakly positive subgroup, *RdRp* gene Cq values showed a strong correlation between heat-treated and control samples (r=0.749, p=0.000), whereas *N* and *E* gene Cq values showed moderate correlations (r=0.400, p=0.000 and r=0.535, p=0.001, respectively).

## DISCUSSION

The main finding of our study is that heat inactivation at 56 °C for 30 min did not significantly affect SARS-CoV-2 detection with the multi-target GeneFinder assay. Among the 200 samples, only one weakly positive became negative after heating, and the overall positive-to-negative ratio was not significantly altered (χ^2^=0.01, p=0.91). Detection of individual targets (*RdRp*, *N*, *E*) was not significantly affected, with very strong correlations observed between heat-treated and control samples, particularly for the *N* and *E* genes in the strongly positive subgroup. These findings align with those from the Wuhan University Renmin Hospital (27) and with an Ethiopian cohort study of 188 patients (28), both reporting no significant effect of heat inactivation on detection rates. Notably, the Ethiopian study reported significant variation in the *N* gene amplification (p=0.010, even more pronounced in weakly positive samples, p=0.001), while in our study, heat-treated strongly positive samples showed significantly higher Cq values for the *N* gene. Moreover, in positive samples (Cq≤40), heat treatment led to significantly higher Cq values for the human *RNase* P gene compared to control samples, whereas in the weakly positive samples, only the *E* gene showed significantly lower Cq values than control samples. This may point to gene-specific susceptibility to heat pre-treatment. In the strongly positive subgroup, correlation analysis corroborated these subtleties: the strongest effects concerned the *N* and *E* genes (r=0.972; r=0.951), whereas the Ethiopian study reported the highest correlation for *ORF1ab* (r=0.978).

**Table 1:** Proportion of detected target genes in SARS-CoV-2-positive control and heat-treated samples

**Groups (batches)**	***RdRp* gene, *N* gene, and *E* gene positive n (%)**	***RdRp* gene and *N* gene positive n (%)**	***N* gene and *E* gene positive n (%)**	***N* gene positive n (%)**
Control	66 (59.46)	18 (16.22)	12 (10.81)	15 (13.51)
Heat-treated	63 (57.27)	25 (22.73)	4 (3.64)	18 (16.36)

*E* gene – envelope gene; *N* gene – nucleocapsid gene; *RdRp* gene – RNA-dependent RNA polymerase gene

**Table 2:** Mismatching RT-qPCR results for control and heat-treated SARS-CoV-2-positive samples

**Sample ID**	**Untreated sample result (Cq value)**			**Heat-treated sample result (Cq value)**		
	***RdRp* gene**	***N* gene**	***E* gene**	***RdRp* gene**	***N* gene**	***E* gene**
S 018	Positive (39.37)	Positive (32.86)	Positive (35.50)	Negative	Positive (33.47)	Negative
S 023	Positive (38.29)	Positive (33.25)	Positive (38.73)	Negative	Positive (32.09)	Positive (33.53)
S 024	Negative	Positive (36.13)	Negative	Negative	Positive (32.78)	Positive (34.97)
S 028	Positive (39.76)	Positive (34.92)	Negative	Negative	Negative	Negative
S 033	Negative	Positive (32.93)	Positive (38.15)	Positive (38.00)	Positive (31.29)	Positive (32.69)
S 055	Positive (39.28)	Positive (32.96)	Positive (35.05)	Negative	Positive (33.74)	Negative
S 056	Positive (39.44)	Positive (33.78)	Positive (36.06)	Negative	Positive (31.71)	Positive (34.04)
S 060	Positive (39.56)	Positive (33.54)	Positive (38.39)	Negative	Positive (32.35)	Positive (33.90)
S 061	Positive (39.87)	Positive (34.29)	Negative	Negative	Positive (32.17)	Positive (34.65)
S 065	Negative	Positive (40.0)	Positive (36.29)	Negative	Positive (40.0)	Negative
S 068	Positive (38.71)	Positive (39.44)	Negative	Negative	Positive (38.16)	Negative
S 070	Negative	Positive (32.26)	Positive (38.52)	Positive (37.96)	Positive (31.93)	Positive (32.91)
S 092	Positive (39.63)	Positive (32.63)	Positive (35.96)	Negative	Positive (33.43)	Negative
S 096	Positive (38.59)	Positive (33.73)	Positive (38.54)	Negative	Positive (32.35)	Positive (33.90)
S 098	Negative	Positive (36.29)	Negative	Negative	Positive (32.65)	Positive (34.77)
S 102	Positive (39.92)	Positive (34.18)	Negative	Negative	Positive (40.0)	Negative
S 107	Negative	Positive (32.52)	Positive (38.26)	Positive (37.15)	Positive (31.96)	Positive (32.70)

Cq – quantification cycle; *E* gene – envelope gene; *N* gene – nucleocapsid gene; *RdRp* gene – RNA-dependent RNA polymerase gene

**Table 3:** Paired comparison of mean Cq values between the control and heat-treated SARS-CoV-2-positive samples

	**Mean Cq value (95 % CI)**	**Paired t-test differences**	
		**Mean difference (95 % CI)**	p-value*
human *RNase* P gene (control batch) (n=111)	27.50 (27.12–27.88)	0.653 (0.31–0.99)	**<0.001**
human *RNase* P gene (heat-treated batch) (n=110)	28.16 (27.66–28.65)		
*RdRp* gene (control batch) (n=40)	31.19 (29.88–32.50)	–1.04 (−2.17–0.27)	**0.011**
*RdRp* gene (heat-treated batch) (n=41)	30.15 (28.76–31.54)		
*N* gene (control batch) (n=67)	31.02 (29.90–32.14)	0.61 (0.33–0.88)	**<0.001**
*N* gene (heat-treated batch) (n=60)	31.22 (30.24–32.19)		
*E* gene (control batch) (n=40)	28.93 (27.57–30.29)	–0.65 (−0.15–1.14)	**0.010**
*E* gene (heat-treated batch) (n=41)	28.29 (27.16–29.42)		
Weakly positive samples (Cq>33) (n=252)			
*RdRp* gene (control batch) (n=47)	34.70 (34.09–35.31)	0.11 (−0.60–0.83)	0.751
*RdRp* gene (heat-treated batch) (n=47)	34.82 (33.91–35.73)		
*N* gene (control batch) (n=44)	34.44 (34.00–34.88)	0.15 (−0.44–0.75)	0.612
*N* gene (heat-treated batch) (n=50)	34.59 (33.93–35.25)		
*E* gene control batch) (n=38)	34.95 (34.32–35.58)	–0.93 (−1.73–0.14)	**0.020**
*E* gene (heat-treated batch) (n=26)	34.02 (33.39–34.65)		

* significant differences are in boldface; CI – confidence intervals; Cq – quantification cycle;*E* gene – envelope gene; IC – internal control; *N* gene – nucleocapsid gene; *RdRp* gene – RNA-dependent RNA polymerase gene

While many studies report negligible effects like ours does (21, 27, 29), some reports indicate that elevated temperatures may degrade viral RNA and thus increase Cq, rendering weak positive or even false negative findings, particularly in low viral load samples (Cq>35) (22, 30, 31, 32). As noted above, these discrepancies likely reflect different susceptibility to heat between viral strains, but the differences may also be owed to different specimen types, reagents, and cohort size.

In general, however, the current body of evidence suggests that heat inactivation at 56 °C for 30 min does not impair SARS-CoV-2 detection by RT-qPCR, although gene-specific sensitivity variations and the risk of false negatives in low viral load samples must be taken into account.

Our study has several limitations. We analysed only NP and OP swabs, excluding other specimen types such as blood, saliva, sputum, or stool, which limits the generalisability of our findings. We also evaluated a single heat inactivation protocol (56 °C for 30 min) and a single commercial RT-qPCR assay, which limits extrapolation to alternative protocols or platforms. Moreover, our analysis was limited to the qualitative detection of viral RNA. We did not quantify the number of RNA copies to establish these differences between heat-treated and control samples, did not assess RNA degradation, nor did we compare heat treatment with chemical inactivation methods. Finally, the observed effect of heat inactivation was most pronounced in samples with low viral load (evidenced by high Cq), which increases the risk of false negatives.

## CONCLUSION

Regardless of these limitations, our findings show that heat inactivation at 56 °C for 30 min does not significantly affect the overall detection rate of SARS-CoV-2 with RT-qPCR. However, it led to changes in the Cq values of targeted viral genes, including target loss, gain, and, in some cases, complete signal disappearance, most notably in weakly positive samples (Cq>33). Even so, heat inactivation at 56 °C for 30 min offers a reasonable compromise between laboratory biosafety and diagnostic integrity when using the multi-target GeneFinder assay.

To minimise diagnostic error, we believe that rigorous pre-analytical control and validated multi-target RT-qPCR protocols remain essential. Further validation across a broader range of specimen types, alternative inactivation protocols, and quantitative methodologies is warranted to establish standardised guidelines to optimise both protocol safety and sensitivity.
